# The knowledge domain and emerging trends in apple detection based on NIRS: A scientometric analysis with CiteSpace (1989–2021)

**DOI:** 10.1002/fsn3.3010

**Published:** 2022-08-24

**Authors:** Xueting Ma, Huaping Luo, Jiean Liao, Jinfei Zhao

**Affiliations:** ^1^ Modern Agricultural Engineering Key Laboratory at Universities of Education Department of Xinjiang Uygur Autonomous Region Tarim University Alar 843300 China; ^2^ College of Mechanical and Electrical Engineering Tarim University China

**Keywords:** apple detection, CiteSpace, NIRS, visual analysis

## Abstract

In this paper, 317 literature in the Web of Science (WoS) related to research on apple by near‐infrared spectroscopy (NIRS) were drawn on the knowledge map of the number of literature, the co‐occurrence network of authors and institutions, the co‐occurrence and clustering of keywords based on CiteSpace. And a related analysis was carried out. Combined with the results of visual analysis and related literature, the research hotspots were sorted out and discussed. This paper provides a certain reference for relevant researchers to study in this field and provides a new method for macroscopically grasping the current status of apple quality detection research, which helps new researchers to quickly integrate into this field and obtain more valuable scientific information.

## Graphical abstract

As many as 317 literature in the Web of Science (WoS) related to research on apple by near‐infrared spectroscopy (NIRS) were drawn on the knowledge map of the number of literature, the co‐occurrence network of authors and institutions, the co‐occurrence and clustering of keywords based on CiteSpace. And a related analysis was carried out.

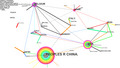

## 1 INTRODUCTION

Apple is one of the most important fruit species in the world, and it is also one of the most consumed fruits. It is popular because of its unique taste and rich nutrients. In China, the United States, Turkey, and other countries, the annual output of apples has increased steadily. In 2020, the output of apples in China reached 40,501,041 tons (data sourced from The Food and Agriculture Organization Corporate Statistical Database (FAOSTAT)). With the development of the global economy, consumers are increasingly demanding high‐quality apples, and traditional inefficient and destructive testing methods are no longer suitable for contemporary needs. In recent years, many scholars have begun to explore better detection methods. Among them, near‐infrared spectroscopy (NIRS) technology has been widely valued and applied in many fields, especially in the agricultural field, due to its nondestructive, fast, efficient, simple, and pollution‐free advantages (Malvandi et al., 2022; Zhang, Jiang, et al., 2022).

The NIRS refers to electromagnetic waves with wavelengths between the visible and midinfrared regions, with a wavelength range of about 780–2500 nm. The self‐vibration of the organic functional groups (OH, CH, NH, SH) in the sample absorbs the energy of the corresponding wavelength in the near‐infrared spectral region, resulting in energy transition and manifested in the spectrum (Lin et al., 2019). The NIRS technology comprehensively uses the latest research results of computer technology, chemometrics, and other disciplines (Williams, 2020), and is widely used in various research fields including apple testing. After years of development, the technology has also achieved many results in the field of apple testing, and its application theory has become more and more mature, such as apple variety identification (Li et al., 2018), sugar acidity detection (Tian et al., 2020), and external damage detection (Nturambirwe et al., 2020).

With the increasing application of NIRS technology in the field of apple detection, and the maturity of technology, it is necessary to synthesize the current state of knowledge and clarify the evolution of this field and its future development direction. The traditional review is mainly based on the induction and summary of relevant literature, sorting out the research results and progress, the research direction is relatively single, only the macroscopic qualitative description and revealing of certain laws and conclusions, although these existing reviews are very valuable for scholars to understand the field. However, they mainly rely on qualitative methods to review the content and themes of the existing literature, and it is difficult to comprehensively and objectively reflect the whole picture of the research field, and it is difficult to systematically show the development process of the research field.

As a research method in the fields of scientometrics and informetrics, a scientific knowledge graph can not only reveal the source of knowledge and the law of development, but also reveal the structural relationship and evolution law of knowledge in related fields in the form of graphical expression (Chen et al., 2021). Based on CiteSpace software, this study describes the distribution characteristics of publications, the international collaboration of countries/regions, the co‐occurrence of subject categories, and the evolution of research hotspots using bibliometric and scientometric methods. These results may help new researchers quickly integrate into the field of apple detection based on NIRS, as they can easily grasp the frontiers of apple detection based on NIRS research and obtain more valuable scientific information. This study also provides references for the follow‐up research of relevant researchers.

## 2 DATA SOURCES AND RESEARCH METHODS

### 2.1 Data sources

This article uses the Web of Science (WoS) database as the data source, and the search deadline is December 31, 2021. The WoS database search selects “Web of Science Core Collection,” and inputs in the search formula: TS = (‘near‐infrared spectroscopy’ OR ‘NIR*’) AND TI = (apple), the retrieved results were further screened by language (select English), document type (select review and paper), etc. With the help of CiteSpace data deduplication function, 317 documents were finally obtained.

### 2.2 Research methods

CiteSpace software is a visual analysis software based on bibliometrics. It needs to run in the Java environment. It can analyze relevant information in a large number of documents (such as the publication and cooperation of authors and institutions, keyword co‐occurrence and clustering, national cooperation, etc.) which is displayed in a visual form, and the relevant information of a certain research field is selectively presented on the map according to our needs, so that researchers can find effective information from it, and intuitively analyze the research development context and hotspots and trends in this research field (Zhang et al., 2020). This study is based on this software to conduct a review of apple detection research based on NIRS.

## 3 RESEARCH RESULTS AND ANALYSIS

### 3.1 Literature published analysis

**FIGURE 1:**
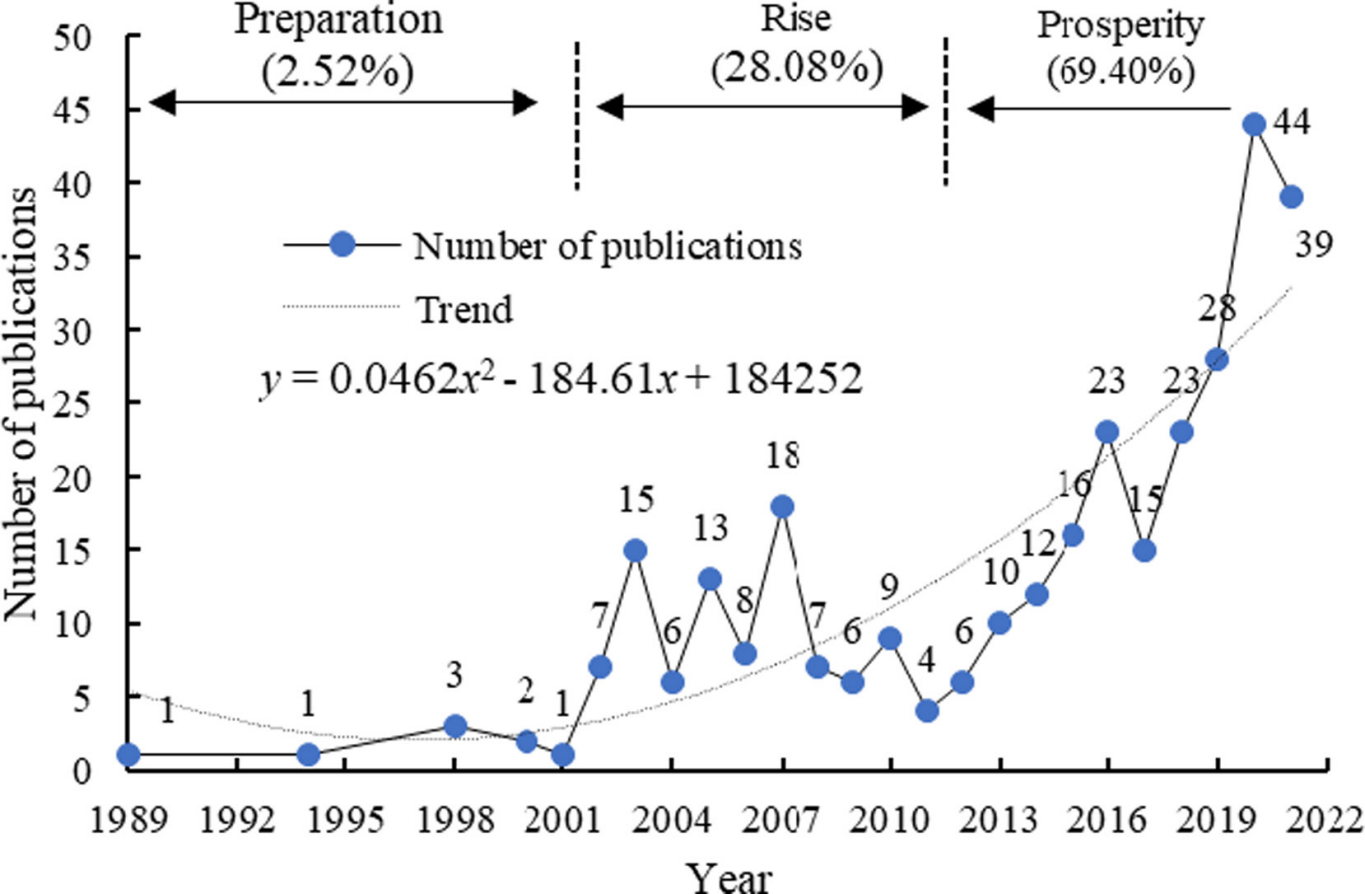
The annual number of literature concerning apple detection based on near‐infrared spectroscopy (NIRS) from 1989 to 2021

The number of published literature is an important indicator for evaluating the development process of this field. Draw a line graph for the number of published literature counted by CiteSpace (Figure 1). Published statistics show that apple detection literature based on the NIRS technology appeared as early as 1989. The literature is *A New Mathematical Procedure for Nir Analysis ‐ the Lattice Technique ‐ Application to the Prediction of Sugar Content of Apples* published by Robert P in *Applied Spectroscopy*. The main content of this literature is to obtain near‐infrared spectral data of apples through near‐infrared spectral detection technology and establish a prediction model to successfully predict the sugar content of apples (Robert et al., 1989).

The publication trend of apple detection literature based on NIRS has roughly experienced a slow development stage (1989–2001), a steady growth stage (2002–2010), and a significant growth stage (2011–2021). From 1989 to 2001, the number of published literature was very small, with an average of less than 1 literature published per year, and the number of published literature at this stage only accounted for 2.52% of the total, indicating that apple detection based on NIRS technology has just started at this stage. From 2002 to 2010, the number of published literature increased, with an average of 10 literature per year, accounting for 28.08% of the total in this stage, indicating that with the further development of computer technology and spectroscopy technology in this stage, the output of apples has been further improved, more and more attention has been paid to the research on apple detection. From 2011 to 2021, the number of published literature showed a trend of significant increase. The number of published literature at this stage accounted for 69.40% of the total. This was mainly due to the significant increase in apple production and the further improvement of people's economic level. More and more attention is paid to the quality of fruits represented by apples. In 2020, the number of published literature reached a maximum of 44. According to statistics, the top three countries in apple production, namely China, the United States, and Turkey, have apple production of 40,501,041, 4,650,684, and 4,300,486 (unit: ton). The above trends in the number of published literature reflect that many scholars are paying more and more attention to research in this field, which is consistent with the development law of the apple industry worldwide, reflecting the increasing demand for apple detection.

### 3.2 Author cooperation network analysis

**FIGURE 2:**
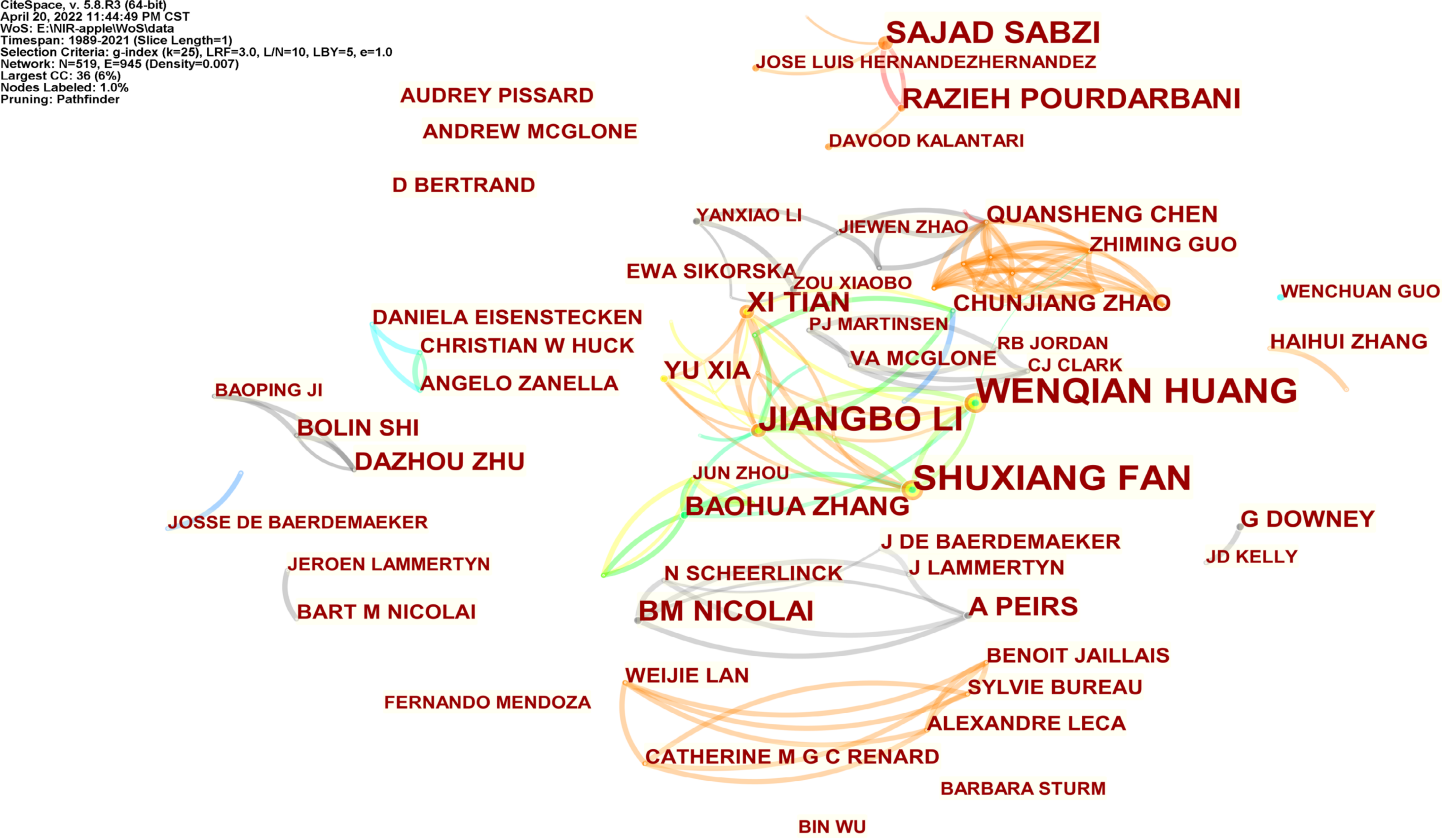
Author collaboration network

The author collaboration network graph can reflect the core authors in the research field and their collaboration and mutual citation relationships (Chu et al., 2022). Based on the author analysis function of CiteSpace, the cooperative network and relevant author information in the fruit detection field are obtained (Figure 2, Table 1). Each node represents an author, and the size of the node. The connection between nodes, and the width, respectively, represent the amount of published literature, the cooperation relationship, and the strength between the authors of the published literature. Figure 2 shows a total of 519 and 945 cooperation lines, with a density of 0.007. Some researchers in this field have formed stable teams and cooperated relatively closely. For example, Shuxiang Fan, Wenqian Huang, Jiangbo Li, and the like, have maintained a state of close cooperation. The authors who are tied for the first place in the number of published literature are Shuxiang Fan and Wenqian Huang who are both from China, with 15 pieces of literature each. Table 1 shows that most of the top 20 authors are from China, indicating that Chinese scholars attach more importance to the research on apple detection based on NIRS, which is related to the growing strength of Chinese industry. In addition, there are also authors from Iran, Belgium, and France, who also publish many pieces of literature. Although Chinese authors have a large number of related literature, they studied this field late. For example, the top three Chinese authors only started to engage in research in this field in 2016, while Bart Nicolai and Ann Peirs from Belgium have already carried out research in this field since 2001.

**TABLE 1:** Statistics of top 20 authors on apple detection by near‐infrared spectroscopy (NIRS)

No.	Author	Year	Country	Count
1	Shuxiang Fan	2016	China	15
2	Wenqian Huang	2016	China	15
3	Jiangbo Li	2016	China	14
4	Sajad Sabzi	2020	Iran	10
5	Xi Tian	2019	China	8
6	Razieh Pourdarbani	2020	Iran	8
7	Bart Nicolai	2001	Belgium	8
8	Ann Peirs	2001	Belgium	7
9	Baohua Zhang	2016	China	7
10	Yu Xia	2019	China	6
11	Dazhou Zhu	2007	China	6
12	Bolin Shi	2007	China	5
13	Quansheng Chen	2008	China	5
14	Gerard Downey	2002	Belgium	5
15	Chunjiang Zhao	2014	China	5
16	Catherine Renard	2020	France	4
17	Ewa Sikorska	2016	Poland	4
18	Weijie Lan	2020	China	4
19	Haihui Zhang	2016	China	4
20	Sylvie Bureau	2020	France	4

### 3.3 Institutional cooperation network analysis

**FIGURE 3:**
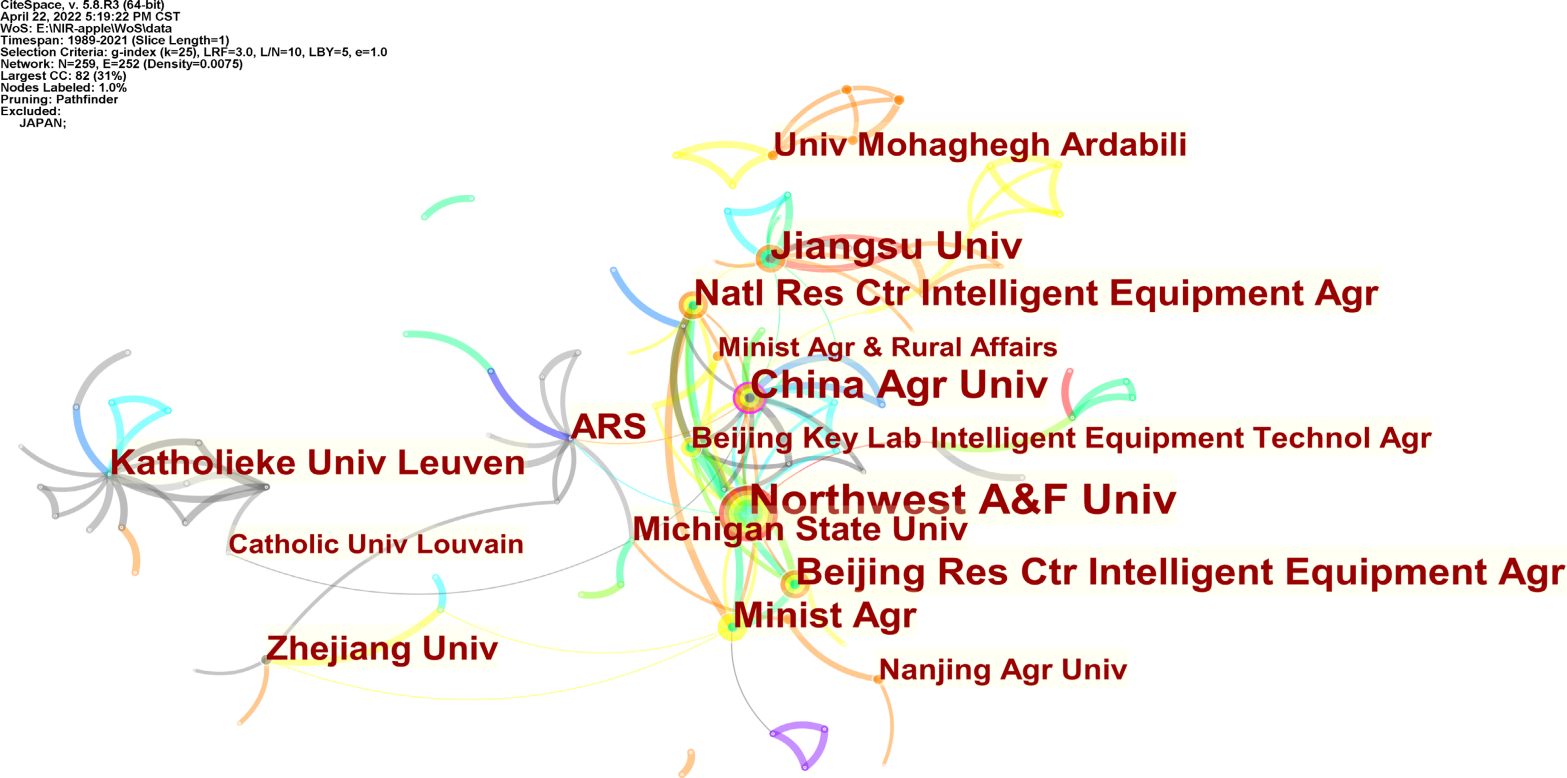
Institutions cooperation network

Among the top 20 institutions by the number of literature, Katholieke Universiteit Leuven's research in this field was earlier, having published literature in 1998. In terms of the number of literature published by institutions, Northwest A&F University ranked first, with 24 literature published, followed by China Agricultural University, Katholieke Universiteit Leuven and Jiangsu University, with 19, 19 and 17 literature, respectively. Most of the top 20 institutions are from China, which shows that Chinese research work in this field is dominant. As far as betweenness centrality is concerned, China Agricultural University has the highest value, indicating that the institution has a greater influence in the apple detection field based on NIRS. The institutions listed in the table all have cooperative relations with other institutions, especially Northwest A&F University and China Agricultural University, which form the core strength of scientific research and have close cooperation with many institutions (Figure 3, Table 2).

**TABLE 2:** Statistics of the top 20 issuing organizations on near‐infrared spectroscopy‐based (NIRS‐based) apple detection

Ranking	Year	Institution	Count	Percentage (%)	Centrality
1	2014	Northwest A&F University	24	7.57	0.05
2	2007	China Agricultural University	19	5.99	0.12
3	1998	Katholieke Universiteit Leuven	19	5.99	0.07
4	2007	Jiangsu University	17	5.36	0.04
5	2016	Beijing Research Center of Intelligent Equipment for Agriculture	16	5.05	0.01
6	2011	National Research Center of Intelligent Equipment for Agriculture	14	4.42	0.02
7	2011	Ministry of Agriculture	14	4.42	0.03
8	2000	Michigan State University	12	3.79	0.09
9	2005	Zhejiang University	11	3.47	0.01
10	2002	Agricultural Research Service	11	3.47	0.04
11	2019	University of Mohaghegh Ardabili	10	3.15	0
12	2016	Beijing Key Lab Intelligent Equipment Technol Agr	8	2.52	0
13	2017	Nanjing Agricultural University	8	2.52	0
14	2019	Ministry of Agriculture and Rural Affairs	6	1.89	0
15	2014	New Zealand Inst Plant & Food Res Ltd	5	1.58	0
16	2002	TEAGASC	5	1.58	0
17	2002	Nagoya University	5	1.58	0.02
18	2006	Washington State University	5	1.58	0
19	2019	Shaanxi Key Lab Agr Informat Percept & Intelligen	5	1.58	0
20	2013	East China Jiaotong University	5	1.58	0

### 3.4 National cooperation network analysis

**FIGURE 4:**
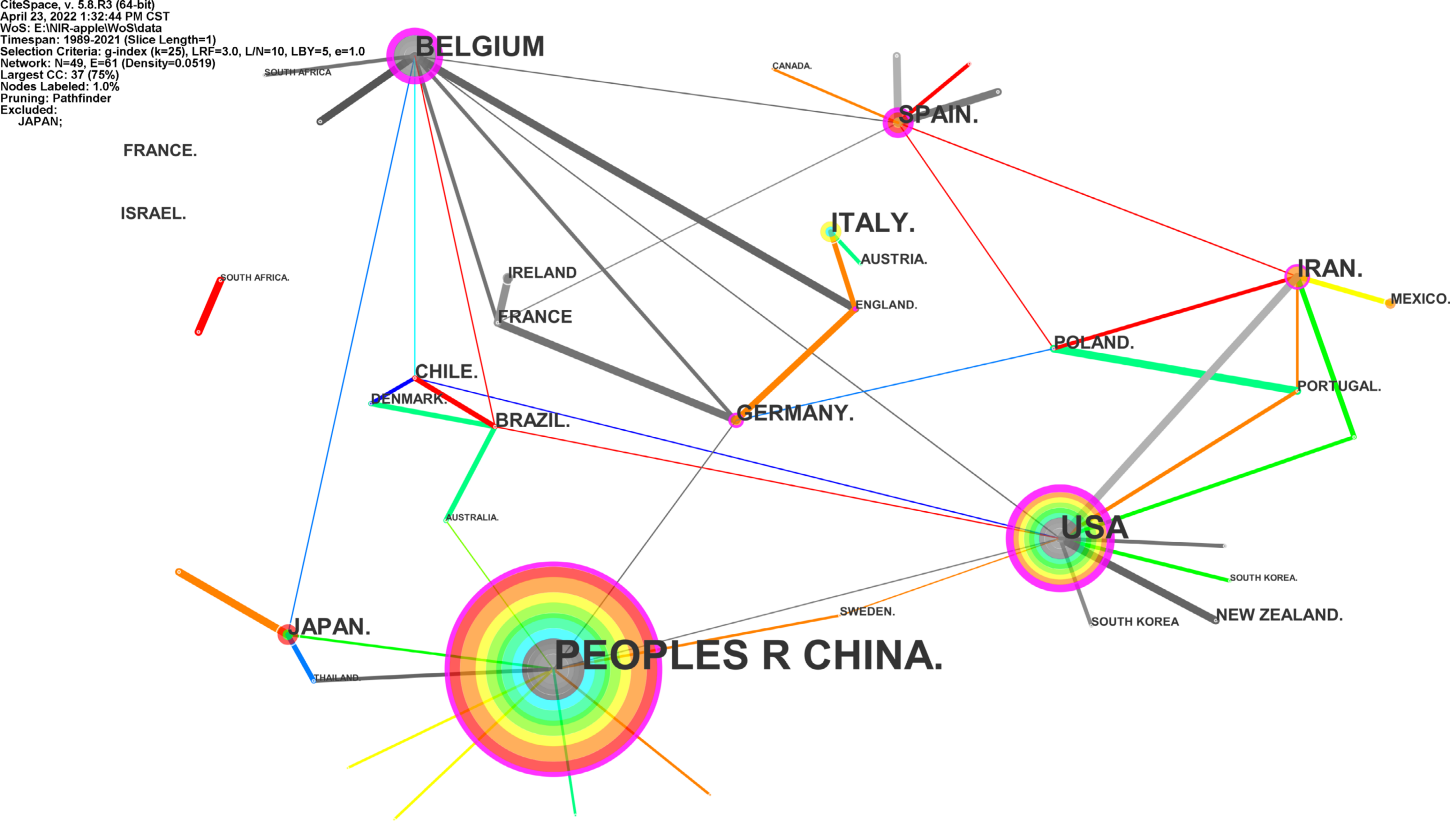
National cooperation network

Based on relevant literature selected from the WoS database, the national cooperation network map and statistical table were drawn through CiteSpace (Table 3, Figure 4). The international apple detection research force based on NIRS comes from 49 countries (regions), mainly in Asia, North America, and Europe, which is consistent with the spatial distribution pattern of apple origin; in terms of research relations, a cooperation network with China, the United States, Belgium, and Spain as the core has been formed. Among them, China published the largest number of literature (127), indicating that China has attached more importance to apple quality detection in the past few decades, which has a certain relationship with the significant increase in Chinese apple production. It is followed by the United States, Belgium, and Spain, with 46, 28, and 21 literature, respectively. Based on the year of first publication, the United States, Belgium, Spain, and Iran were the earlier countries to study this field. Although China publishes the most literature, its centrality (0.32) is significantly lower than those of the United States (0.43) and Belgium (0.43), which indicates that the quality of Chinese scientific research needs to be improved, and there is still room for improvement in exchanges and cooperation with other countries.

**TABLE 3:** Statistics of the top 10 countries that published literature on near‐infrared spectroscopy (NIRS) apple detection

Ranking	Country	Count	Percentage (%)	Year	Centrality
1	China	127	40.06	2005	0.32
2	USA	46	14.51	1994	0.43
3	Belgium	28	8.83	1998	0.43
4	Italy	21	6.62	2008	0.06
5	Spain	16	5.05	1998	0.27
6	Iran	15	4.73	1998	0.12
7	Japan	13	4.10	2008	0.1
8	Germany	11	3.47	2003	0.13
9	Chile	8	2.52	2013	0.03
10	Brazil	8	2.52	2010	0.06

## 4 NIRS‐BASED APPLE DETECTION RESEARCH HOTSPOTS ANALYSIS

### 4.1 Keyword co‐occurrence analysis

**FIGURE 5:**
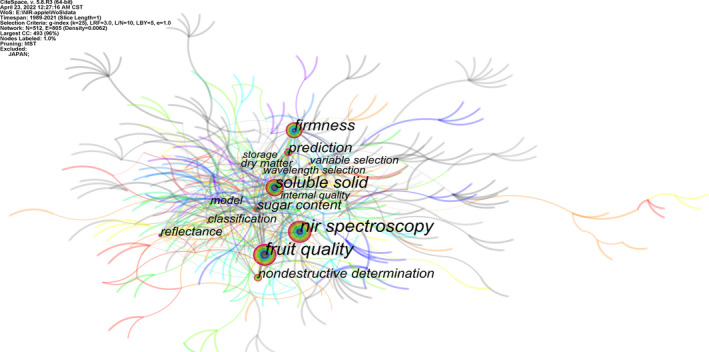
Keyword co‐occurrence network

Keywords carry the most important and core information of the literature, and are the key to grasping the important information in the literature. Therefore, we can understand the research hotspots in a certain field by analyzing the keywords with high frequency (Zheng et al., 2020). Use CiteSpace to analyze the keywords of apple detection research literature based on NIRS, set the Node Type to Keyword, the threshold to *T* = 30, and the rest to default. Eliminate invalid keywords and combine multiple similar keywords, and finally, the research hotspot knowledge map shown in Figure 5 and the top 30 keyword information shown in Table 4 are obtained. The research hotspots of apple detection based on NIRS technology are preliminarily analyzed based on high‐frequency keywords. These keywords come from the title, abstract, author keywords, and keywords provided by WoS. The size of the nodes indicates the frequency of the keywords, and the connection between the nodes reflects the co‐occurrence strength and relationship of the keywords. The larger the node, the higher the frequency of keyword occurrence, and the thickness of the connection line indicates the strength of co‐occurrence between keywords. Figure 5 and Table 4 show that the keywords that appear frequently in the WoS database on apple detection research based on NIRS include quality, soluble solids, firmness, prediction, sugar content, nondestructive determination, reflection, classification, model, dry matter, variable selection, etc. The frequency of these keywords is more than 20 times, indicating that researchers have paid attention to this research field, which can roughly reflect the research hotspot of apple detection based on NIRS

**TABLE 4:** Statistics of top 30 keywords related to near‐infrared spectroscopy (NIRS)‐based apple detection

Keyword	Year	Centrality	Count	Keyword	Year	Centrality	Count
Nir spectroscopy	1998	0.14	181	Bruise detection	1994	0.06	15
Quality	2000	0.34	165	Algorithm	2000	0.06	15
Soluble solids	2000	0.17	118	Calibration	2003	0.18	14
Firmness	2001	0.14	87	Diffuse reflectance	2007	0.08	10
Prediction	2007	0.21	59	System	2000	0.09	10
Sugar content	1998	0.24	47	Chlorophyll	2006	0.06	10
Nondestructive determination	2000	0.18	44	Identification	2003	0.04	9
Reflectance	1998	0.28	33	Hyperspectral imaging	2002	0.07	9
Classification	2005	0.12	27	Brownheart	2009	0.02	8
Model	2005	0.09	24	Cultivar	2005	0.03	8
Dry matter	2002	0.1	22	Regression	2011	0.03	8
Variable selection	2015	0.1	21	Apple juice	2005	0.03	8
Wavelength selection	2007	0.04	19	Discrimination	2009	0.05	8
Internal quality	2005	0.06	17	Time	2016	0.03	7
Storage	2014	0.1	16	Computer vision	2018	0.02	7

### 4.2 Keyword cluster analysis

Based on the knowledge graph of keyword co‐occurrence analysis, cluster analysis is performed on literature data, log‐likelihood ratio (LLR) algorithm is used to extract label words, and similar clusters are merged to show the research hotspot of apple detection based on NIRS technology. The results of cluster analysis of the co‐word knowledge graph are shown in Table 5. Size is the number of keywords contained in the cluster, and only some keywords are listed in the table; Silhouette is an index to measure the homogeneity of the entire cluster members. The larger the value, the more similar the cluster members are high; Mean year represents the average year of the documents in the cluster. According to the clustering results in Table 5, combined with the content analysis of the literature in each cluster, the research hotspots of apple detection based on NIRS technology mainly include the following aspects: (1) Internal quality detection of apple based on NIRS. (2) External quality detection of apple based on NIR. (3) Disease detection of apple based on NIRS.

**TABLE 5:** Keyword clustering information statistics

Size	Silhouette	Mean year	Typical clustering results (LLR)	Cluster label	Secondary manual naming
53	0.735	2010	Apple flesh; ripening; dry matter; nondestructive; firmness	Apple flesh	Internal quality detection of apple based on NIRS
49	0.856	2008	Bruise detection; multispectral; shape‐from‐shading; food safety; image processing	Bruise detection	External quality detection of apple based on NIR
37	0.856	2012	Bitter pit; malus x domestica cripps pink; nondestructive testing; plant nutrition; internal quality	Bitter pit	Disease detection of apple based on NIRS

### 4.3 Subject co‐occurrence analysis

**FIGURE 6:**
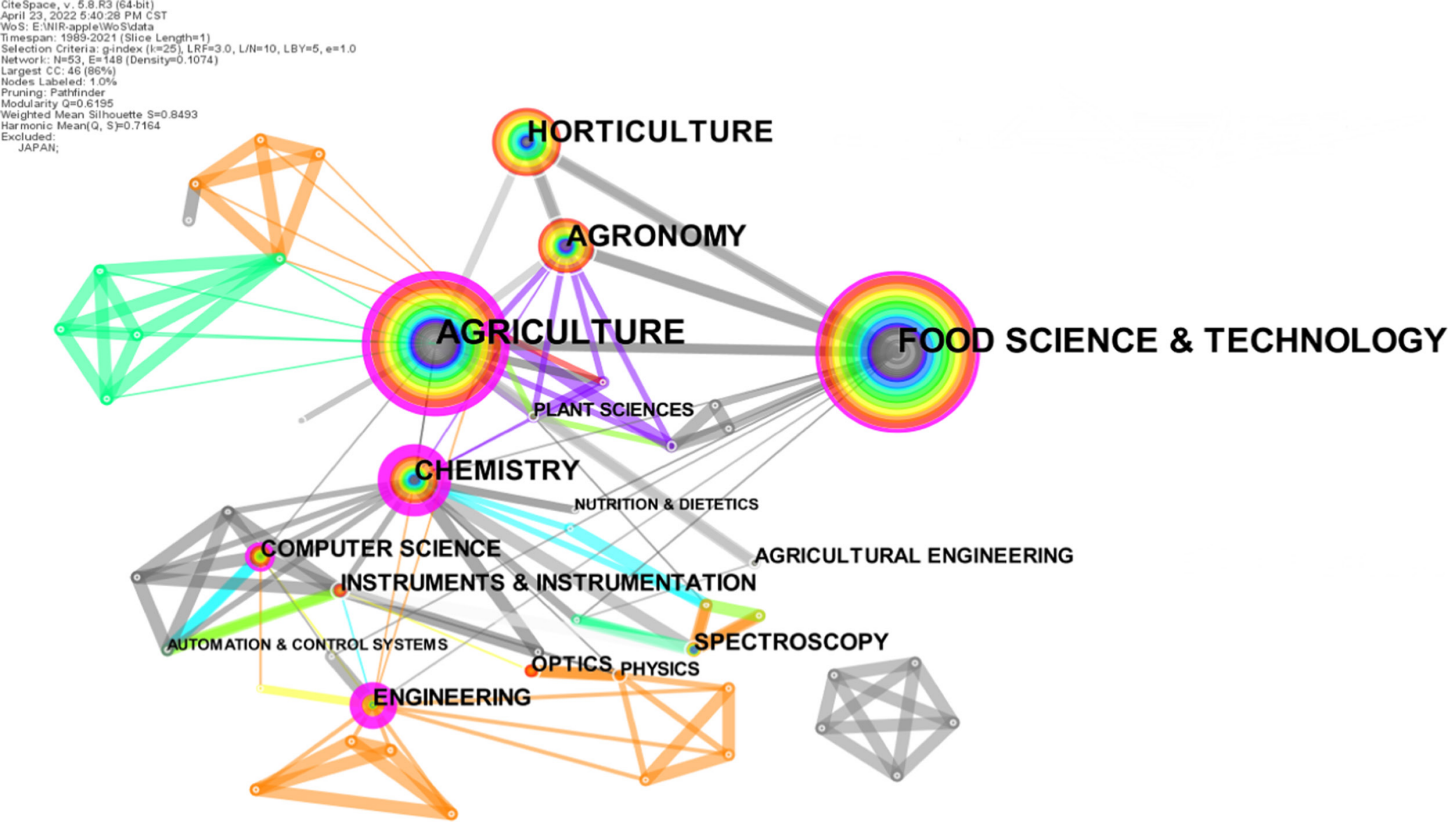
Subject co‐occurrence network

Subject co‐occurrence analysis was performed on all data. Subject co‐occurrence maps and statistical tables were drawn (Figure 6, Table 6). Among them, Food Science & Technology has the largest node, the highest frequency, and an earlier appearance, revealing that NIRS‐based apple detection is mainly based on this discipline. It has a high influence, which may be because the apple detection technology based on NIRS needs to correlate with a certain parameter obtained by the chemical detection method.

**TABLE 6:** Subject information statistics

Ranking	Count	Centrality	Category	Year
1	145	0.2	Food Science & Technology	1998
2	134	0.4	Agriculture	1994
3	69	0	Horticulture	1998
4	65	0.03	Agronomy	1998
5	59	0.47	Chemistry	2002
6	31	0.46	Engineering	2004
7	29	0.1	Instruments & Instrumentation	1989
8	28	0.04	Spectroscopy	1989
9	27	0.11	Computer Science	2005
10	21	0	Agricultural Engineering	1994
11	20	0	Optics	2002
12	15	0.05	Physics	2010
13	14	0.05	Plant Sciences	1998
14	8	0	Nutrition & Dietetics	2011
15	8	0	Automation & Control Systems	2007

### 4.4 Reference analysis

In bibliometric analysis, co‐citation analysis can be used to analyze the composition of the knowledge base of a research field. Table 7 lists the top 10 most cited literature in the research field of apple detection based on NIRS. These highly cited pieces of literature have important implications for this research field. Among them, the most frequently cited literature is *NIR spectroscopy for the optimization of postharvest apple management* published by Giovanelli et al. (2014), in which researchers assessed apple fruit quality by NIRS and monitored postharvest quality changes. Based on partial least squares (PLS) regression and linear discriminant analysis (LDA) classification techniques, a prediction model for apple chemical and physical parameters was established. It shows that NIRS has great potential in estimating apple storage time (Giovanelli et al., 2014). This is followed by Liu et al.'s literature *Color compensation and comparison of shortwave near infrared and long wave near infrared spectroscopy for determination of soluble solids content of ‘Fuji’ apple* published in 2016, which uses a new color compensation method with whose help shortwave near‐infrared spectroscopy (SWNIR) and long‐wave near‐infrared spectroscopy (LWNIR) for prediction of apple soluble solids content were compared and linear and nonlinear regression models were considered. It was shown that color compensation can significantly improve the prediction performance of SWNIR models (Guo et al., 2016). In addition, in the literature *Effect of spectrum measurement position variation on the robustness of NIR spectroscopy models for soluble solids content of apple* published by Fan et al. (2016), the researchers studied the effect of spectral measurement position variation on the detection of apple soluble solids (soluble solids content (SSC)). SSC compensation models were built separately for each measured location (local location model) and the full dataset containing all locations (global location model) using partial least squares (PLS). The results show that the measurement location affects the acquired spectral information, which in turn affects the prediction accuracy of SSC. Compared with the local position model, the global position model has higher prediction accuracy (Fan et al., 2016). From the important references shown in Table 7, it can be seen that postharvest quality monitoring of apples, detection of internal components represented by soluble solids, and establishment of apple quality prediction models are the research areas in this field. The results are consistent with the results obtained by cluster analysis.

**TABLE 7:** Top key literature for research on apple detection using near‐infrared spectroscopy (NIRS)

Frequency	Centrality	Year	Literature
27	0.07	2014	NIR spectroscopy for the optimization of postharvest apple management Giovanelli et al. (2014)
24	0.11	2016	Color compensation and comparison of shortwave near infrared and long wave near infrared spectroscopy for determination of soluble solids content of ‘Fuji’ apple Guo et al. (2016)
20	0.03	2016	Effect of spectrum measurement position variation on the robustness of NIR spectroscopy models for soluble solids content of apple Fan et al. (2016)
16	0.45	2010	Postharvest quality of apple predicted by NIR‐spectroscopy: Study of the effect of biological variability on spectra and model performance Bobelyn et al. (2010)
14	0.03	2019	Long‐term evaluation of soluble solids content of apples with biological variability by using near‐infrared spectroscopy and calibration transfer method Fan et al. (2019)
11	0.02	2014	Grading of apples based on firmness and soluble solids content using Vis/SWNIR spectroscopy and spectral scattering techniques Mendoza et al. (2014)
11	0.09	2003	Effect of biological variability on the robustness of NIR models for soluble solids content of apples Peirs et al. (2003)
10	0.24	2014	Automatic sample rotation for simultaneous determination of geographical origin and quality characteristics of apples based on near infrared spectroscopy (NIRS); Schmutzler and Huck (2014)
9	0.01	2020	Quantitative detection of apple watercore and soluble solids content by near infrared transmittance spectroscopy Guo et al. (2020)
9	0.16	2013	Non‐destructive measurement of vitamin C, total polyphenol and sugar content in apples using near‐infrared spectroscopy Pissard et al. (2013)

## 5 DISCUSSION

According to the visual analysis results of apple detection research based on NIRS technology, the research focuses mainly on NIRS‐based apple internal quality detection, NIRS‐based apple external quality detection, and NIRS‐based apple disease detection in this field. No matter which aspect of research, it is mainly based on the rule that different quality apples have different internal components and external characteristics, and will have different absorption and reflection characteristics under different wavelengths of light (Anderson & Walsh, 2022; Pourdarbani et al., 2022). That is to say, the spectral reflectance or absorptivity of apples in a certain wavelength is larger than that of other parts. According to this characteristic, combined with the optical detection device, the nondestructive detection of apple quality can be realized (Qin et al., 2021). The key steps of apple detection based on NIRS are spectral preprocessing and model establishment. Common spectral pretreatment methods include standard normal distribution (SNV), multiplicative scatter correction (MSC), Savitsky–Golay (S–‐G), de‐trend, Smoothing (S), Derivative (D), and so on. Commonly used algorithms for modeling include multiple linear regression (MLR), partial least squares (PLS), artificial neural network (ANN), support vector machine (SVM), and so on. Novel algorithms like the firefly algorithm (FA), improved particle swarm optimization‐extreme learning machine (IPSO‐ELM), etc., have appeared in recent years (Anderson & Walsh, 2022; Bobelyn et al., 2010; Fan et al., 2019; Zhang, Huang, et al., 2022).

### 5.1 Research on apple's internal quality detection based on NIRS

The internal quality detection of apples mainly includes the detection of chemical components. The chemical components include the detection of soluble solids, acidity, vitamins, starch, etc., of which there are relatively many studies on soluble solids content (SSC) and acidity. SSC and acidity are important factors that affect the taste of apples. Since different ripenesses of apples correspond to different acidity values, acidity detection can also be used to judge the ripeness of apples (Pourdarbani et al., 2022). Table 8 is a case study of apple's internal quality inspection based on NIRS. The practice has proved that for the detection of apple soluble solids and acidity, the prediction model established by PLS has a good effect, and high accuracy, and is widely used.

**TABLE 8:** Study on apple soluble solids content (SSC) and acidity detection based on near‐infrared spectroscopy (NIRS)

Cultivars	Test items	Main method	Model effect	Literature sources
Fuji	SSC	MSC + SNV + S‐G + IPSO‐ELM	𝑅^2^ = 0.993, RMSEP = 0.0155, RPD = 11.6	Zhang, Huang, et al. (2022)
Fuji	SSC	S + SNV+ D + PLS	𝑅^2^ = 0.777, RMSEP = 0.0056, RPD = 2.11	Fan et al. (2020)
Fuji	SSC	S‐G + SNV + PLS	𝑅^2^ = 0.87, RMSEP = 0.59	Zhang, Zhang, et al. (2021)
Jinshuai	Acidity	NORM‐SG‐MSC + CARS+PLS	𝑅^2^ = 0.9615, RPD = 5.0987	Zhang, Chen, et al. (2021)
Fuji	Acidity	ANN + FA	𝑅^2^ = 0.86, RMSEP = 0.226	Pourdarbani et al. (2022)

### 5.2 Research on apple external quality detection based on NIRS

The external quality detection of apples mainly includes the detection of external damage, rot, and pesticide residues. Damage and rot are due to the peeling or cracking of the skin due to bumping and squeezing during manual picking, handling or transportation, and further development will eventually lead to discoloration and rot (Mogollon et al., 2020; Nturambirwe et al., 2020; Tang et al., 2020). Pesticide residues are mainly caused by the excessive application of pesticides and the use of unreasonable cleaning methods (Chen et al., 2020). Pang et al. (2022) developed a near‐infrared hyperspectral imaging system, explored three sensitive areas, and selected characteristic wavelengths from the three areas based on the principal component analysis (PCA), established a YOLOv3 (You Only Look Once, Version 3) model, and successfully detected apple damage. Luo et al. (2019) used PCA based on NIRS to detect apple bruise damage, and the overall detection accuracy was 99.5%. Nturambirwe et al. (2020) successfully not only detected bruises of three apple species based on near‐infrared (NIR) imaging technology and partial least squares discriminant analysis (PLS‐DA), but also pointed out that the fruit variety has a certain impact on the bruise detection ability.

### 5.3 Research on apple disease detection based on NIRS

During storage and transportation of apples, various internal diseases such as brown rot, bitter pit, ring rot, moldy core, and watercore may occur (Chang et al., 2020; DeBrouwer et al., 2020; Grabowski, 2021; Sun et al., 2022). Diseases affect the quality of apples, which are often difficult to detect in the early stage of the disease, and some diseases are infectious. Moldy core, which is common, expands outward from the ventricle of the apple until it is severely rotted. Disease prevention and timely removal of diseased fruit are effective measures at present. It is one of the ideal means to establish a more accurate nondestructive disease detection model by NIRS. Some studies are shown in Table 9.

**TABLE 9:** Study on apple disease detection based on near‐infrared spectroscopy (NIRS)

Cultivars	Test items	Main method	Model effect	Literature sources
Fuji	Moldy core	PCA + PLC‐CA	Accuracy = 93.75%	Qin et al. (2021)
Fuji	Moldy core	SPA+SVM	Accuracy = 100%	Zhang, Jiang, et al. (2022)
Fuji	Moldy core	C (T) + SVM/ BP‐ANN	Accuracy = 90.20%	Tian et al. (2019)
Fuji	Watercore	MSC + FD + SVM	Accuracy = 91.67%	Chang et al. (2020)
Fuji	Watercore	SGS‐SNV + LS‐SVM	Accuracy = 98.48%	Zhang, Wang, et al. (2022)

At present, most of the apple detection work based on NIRS is in the laboratory research stage, and NIRS detection equipment is expensive, which is not conducive to popularization in the general population. Our next step should be to develop a portable detection device based on a mature theoretical basis, which is convenient for fruit farmers and other related staff to use.

## 6 CONCLUSION

This paper conducts a visual analysis of the NIRS‐based apple detection research literature from 1989 to 2021 based on CiteSpace. The results show that: (1) the number of published literature on apple detection based on NIRS has shown an increasing trend, indicating that research in this field has been paid more and more attention worldwide; (2) there are a large number of researchers and research institutions studying this field, which are forming several core researchers and core institutions, and most of the top 20 researchers and institutions are from China, indicating that China has done more research in this field; (3) China has the largest number of published literature, but the betweenness centrality value of the United States and Belgium is the largest, indicating that the United States and Belgium have high‐quality scientific research results and cooperate closely with other countries in this field; (4) keyword co‐occurrence, cluster analysis, subject co‐occurrence, and reference analysis show that the research hotspots in this field are NIRS‐based apple internal quality detection, NIRS‐based apple external quality detection, and NIRS‐based apple disease detection. According to the visualization analysis results, the hot research on these three aspects is discussed. This literature provides a certain reference for the relevant personnel of apple detection research based on NIRS, which is beneficial to grasp the research status.

## FUNDING INFORMATION

This study was supported by the funding from the National Natural Science Foundation of China (11964030); the Open Project of Key Laboratory of Modern Agricultural Engineering in Colleges and Universities of the Department of Education of the Autonomous Region (TDNG2021201).

## CONFLICT OF INTEREST

The authors declare no conflicts of interest.

## Data Availability

The data of this study are from WoS and can be reflected in the text.
